# Temporal changes in device-derived daily activity related to ventricular arrhythmias from the CERTITUDE registry

**DOI:** 10.1016/j.hroo.2024.07.020

**Published:** 2024-08-20

**Authors:** Valentina Kutyifa, Michael Christof, Steven Mullane, Camden Harrell, Jagmeet Singh, Larry Chinitz, Niraj Varma, Jonathan P. Piccini, Mintu P. Turakhia, Spencer Z. Rosero

**Affiliations:** 1 Division of Cardiology, Department of Medicine, University of Rochester, Rochester, New York; 2 Division of Cardiology, Biotronik Inc., Lake Oswego, Oregon; 3 Division of Cardiology, Massachusetts General Hospital, Harvard Medical School, Boston, Massachusetts; 4 Division of Cardiology, Heart Rhythm Center, NYU Langone Health, New York, New York; 5 Division of Cardiology, Cleveland Clinic, Cleveland, Ohio; 6 Division of Cardiology, Duke University Medical Center, Durham, North Carolina; 7 Division of Cardiology, Stanford University, Stanford, California

**Keywords:** Ventricular arrhythmias, Physical activity, Therapy, ICD, CRT-D

## Abstract

**Background:**

There have been limited data examining the temporal relationship between device-derived daily activity and ventricular arrhythmias (VAs).

**Objective:**

We aimed to assess whether activity predicted VAs or VAs predicted changes in activity.

**Methods:**

The CERTITUDE registry includes over 55,000 implanted devices active on Home Monitoring. Daily data on activity are captured by a 1-axis accelerometer. Temporal changes in activity during treated VAs were analyzed using the first event and 7-day activity windows (baseline, pre-event, and postevent). Baseline period was defined as 31 to 38 days prior to VA. VAs were categorized by heart rate (≤200 beats/min, >200 beats/min) and treatment (shock or antitachycardia pacing). Differences were assessed using the binomial proportion test and case-crossover analysis.

**Results:**

A total of 5631 devices (3688 implantable cardioverter-defibrillators, 1943 cardiac resynchronization therapy defibrillators) were analyzed with a cumulative follow-up duration of 18,354 years (5.6 million days). Patients with VA events >200 beats/min with shock (n = 593) had a significant decline in activity post-VA with a median –8.7% reduction (interquartile range –24.6% to 7.3%, *P <* 0.001). However, there was no reduction in activity before VA events >200 beats/min (*P =* .690) or before or after VA events >200 beats/min with antitachycardia pacing. However, VA events ≤200 beats/min with shock had reductions in activity following the event (–5.8%, interquartile range –29.5% to 12.3%, *P =* .003). Case-crossover analyses confirmed lower activity rates following for VA events >200 beats/min with shock.

**Conclusion:**

In the CERTITUDE registry, we have shown a temporal decline in device-derived activity following VA events >200 beats/min and for VA events <200 beats/min treated with a shock, but we did not find changes in activity preceding a shock event.

## Graphical abstract

**Figure:**
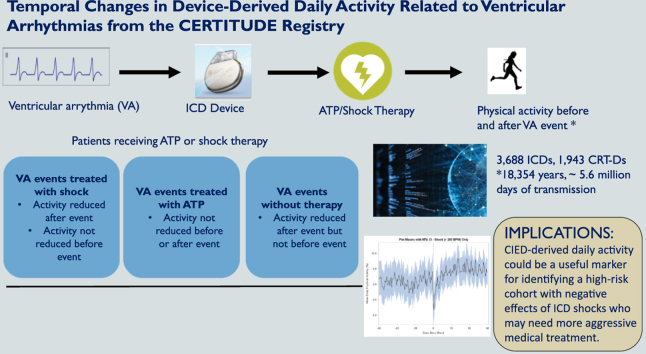

**Key Findings**

▪ In the CERTITUDE registry, we have shown a temporal decline in device-derived physical activity following ventricular arrythmia (VA) events >200 beats/min and for VA events <200 beats/min treated with a shock.
▪ However, we did not find changes in device-derived physical activity preceding a shock event.
▪ There were no significant changes in device-derived physical activity in the 7 days prior to a VA event in the study.
▪ Cardiac implantable electronic device–derived daily activity could be a useful marker for assessing the effects of implantable cardioverter-defibrillator shocks and identify those with need for more aggressive treatment.

## Introduction

Despite recent advances in cardiovascular implantable electronic device (CIED)–based arrhythmia detection, remote monitoring, and therapies, there remains a limitation to precisely predict an individual’s total physiologic response, such as daily activity, especially when related to ventricular arrhythmias (VAs). Arrhythmias originating from both atria and ventricles are highly variable between individuals and often significantly impact daily function and quality of life. Atrial high-rate episodes that are temporally correlated with VAs have been shown to be associated with adverse outcomes.^1^ However, it is not known to what degree VAs, especially those with therapy delivered by implantable cardioverter-defibrillators (ICDs) would affect activity in CIED patients.

Activity has been previously suggested to predict an increased risk for both cancer and cardiovascular mortality.^2^, ^3^, ^4^ In a large-scale population study, researchers successfully analyzed accelerometer-acquired physical activity in the United Kingdom and reported a median wear time of 6.9 days with a target of 7 days with excellent compliance (93.3%).^5^ Matthews and colleagues^6^ described the best practices for using physical activity monitors in population-based research, which further paves the road for this new digital biomarker. Another study reported from 26,509 cardiac resynchronization therapy (CRT) patients followed on remote monitoring and suggested that changes in activity following CRT are linked to clinical outcomes^7^ and mortality.^8^ However, the temporal relationship between activity and VAs has not been studied.

Therefore, this study seeks to leverage modern CIED technologies with daily monitoring to characterize the relationship between VAs and physical activity. We aim to evaluate the temporal relationship between activity and VAs to identify whether changes in activity precede arrhythmias or arrhythmias precede changes in activity.

## Methods

The integration of the multimodal sensing capabilities of implantable CIEDs with remote monitoring facilitates the acquisition of real-world data^9^ to better understand the role of digital biomarkers, such as activity.

The CERTITUDE registry comprises of a de-identified database of over 55,000 U.S. Biotronik pacemaker, ICD, CRT, and loop recorder devices active on Home Monitoring, implanted prior to 2021, and have provided authorization for the use of their data for research purposes.^8^ Individuals from the CERTITUDE registry implanted with a Biotronik ICD or cardiac resynchronization therapy defibrillator (CRT-D) on or after January 1, 2010, were eligible for inclusion in this analysis. Daily data on VAs and physiological parameters such as activity are captured. The Advarra Institutional Review Board reviewed and approved the CERTITUDE registry, which was granted a waiver of informed consent and a full waiver of Health Insurance Portability and Accountability Act authorization, as the research involved no more than minimal risk to the patients and their privacy and the research could not practicably be carried out without the waivers.

### CIED-derived patient activity

Patient activity is reported daily as percentage active during the day, assessed by a 1-axis accelerometer utilizing the z-axis through active implant, which is approximately also the z-axis through the plane of the body. The continuous measurement checks every 2 seconds whether the patient is moving or not. The CIED-derived data variable of activity are reported in this study as percent active on each day. Activity was used as the primary variable analyzed pre- and post-VAs. Analyses were adjusted for age, sex, and device type.

The primary analysis to determine temporal changes in device-derived activity associated with treated VAs was performed using the binomial proportion test. For this analysis, we identified the first event per device and utilized 7-day activity windows at baseline, pre-event, and postevent for the comparison. A 7-day window was utilized as a clinically meaningful window. Baseline period was defined as 31 to 38 days prior to VAs. VAs were categorized by heart rate (≤200 beats/min, >200 beats/min) and treatment (shock with or without antitachycardia pacing [ATP], ATP alone, no therapy).

Additionally, case-crossover analysis was also performed as a sensitivity analysis. In this analysis, we similarly utilized first events and a 7-day activity window as defined previously. The case-crossover analysis was chosen as an alternative method as the onset of potential changes in activity are sudden and short in duration, and in addition, the individual exposure, “change in activity” prior or after VT events, varies within short time intervals. Episodes within 90 days of implantation were excluded, and devices must have had at least 1 transmission during the case and control period to be included in the analyses.

### Statistical analysis

Continuous variables were reported using mean ± SD or median (interquartile range [IQR]), while categorical variables were reported using frequency and percentage. A Wilcoxon signed rank test was used to compare physical activity between time periods, due to lack of normality. A case-crossover analysis was conducted for the case period, the 7-day window prior to the first event, and 4 control periods, weekly windows of 31 to 38 days, 39 to 46 days, 47 to 54 days, and 55 to 62 days prior to the event, using a conditional logistic regression. This analysis was stratified by patient and adjusted for age, sex, and device type. All statistical analyses were conducted using SAS 9.4 (SAS Institute) using a significance level of .05 (95% confidence interval).

## Results

**Figure 1:**
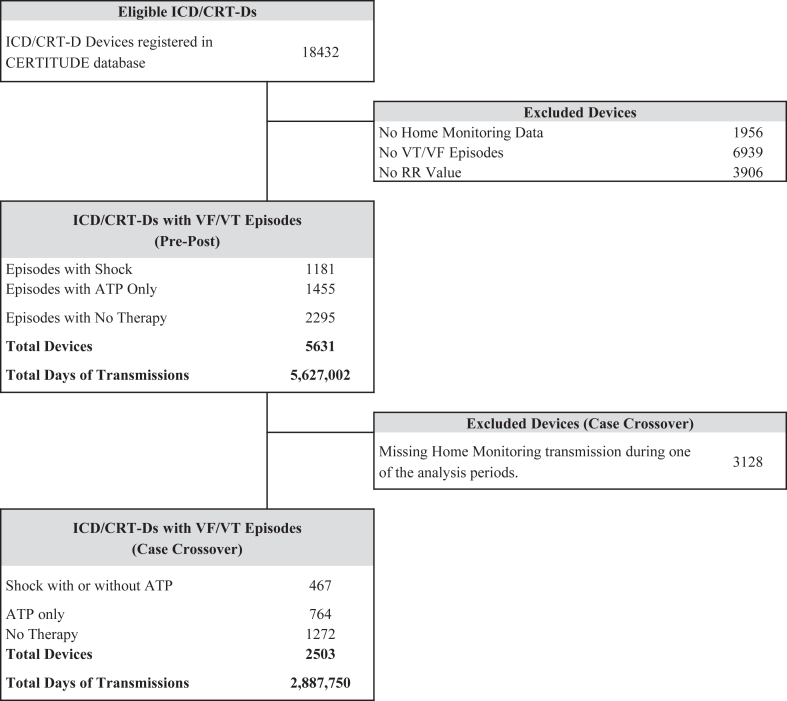
Study population. ATP = antitachycardia pacing; CERTITUDE = •••; CRT-D = cardiac resynchronization therapy defibrillators; ICD = implantable cardioverter-defibrillator; VF = ventricular fibrillation; VT = ventricular tachycardia.

A total of 18,432 Biotronik ICD and CRT-D patient devices were identified from the CERTITUDE registry. Of these devices, 5631 (3688 ICDs and 1943 CRT-Ds) met inclusion criteria and had activity data available for analyses with a cumulative follow-up duration of 18,355 years (5.6 million days with transmission) (Figure 1).

A total of 2636 VA events (1699 VAs >200 beats/min, 937 VAs ≤200 beats/min) were analyzed to evaluate the change in daily activity pre– and post–shock therapy and ATP therapy. Baseline characteristics of the subject cohort and characteristics of patients with VA events ≤200 beats/min vs >200 beats/min with shock or ATP alone are presented in Table 1. The patient age was 65 years on average, 24% were female, 66% had an ICD, and 34% had a CRT-D device.

**Table 1:** Patient demographics

Characteristics	All patients (N = 5631)	1 or more shocks		ATP alone	
		≤200 beats/min (n = 145)	>200 beats/min (n = 1036)	≤200 beats/min (n = 792)	>200 beats/min (n = 663)
Age at enrollment, y	65.2 ± 12.3 (13.7–95.8)	67.5 ± 12.5 (24.4–90.4)	67.3 ± 11.1 (26.8–90.8)	69.6 ± 10.3 (18.4–74.0)	66.6 ± 11.4 (16.0–93.9)
Sex					
Female	1335 (23.7)	24 (16.6)	227 (21.9)	156 (19.7)	121 (18.3)
Male	3854 (68.4)	114 (78.6)	697 (67.3)	578 (73.0)	511 (77.1)
Unknown	442 (7.9)	7 (4.8)	112 (10.8)	58 (7.3)	31 (4.7)
Implant type					
ICD	3688 (65.5)	98 (67.6)	638 (61.6)	459 (58.9)	420 (63.4)
Single chamber	488 (8.7)	8 (5.5)	95 (9.2)	31 (3.9)	54 (8.1)
Dual chamber	1916 934.0)	58 (40.0)	313 (30.2)	305 (38.5)	210 (31.7)
DX	1284 (22.8)	32 (22.1)	230 (22.2)	123 (22.2)	156 (23.5)
CRT-D	1943 (34.5)	47 (32.4)	398 (38.4)	333 (42.1)	243 (36.6)
CRT-DX	25 (0.4)	2 (1.4)	6 (0.6)	0 (0)	6 (0.9)

Values are mean ± SD (range) or n (%).

ATP = antitachycardia pacing; CRT-D = cardiac resynchronization therapy defibrillators; CRT-DX = single-lead cardiac resynchronization therapy defibrillator; DX = single-lead implantable cardioverter-defibrillator system; ICD = implantable cardioverter-defibrillator.

### Primary analysis

#### Changes in activity prior to VAs

**Figure 2:**
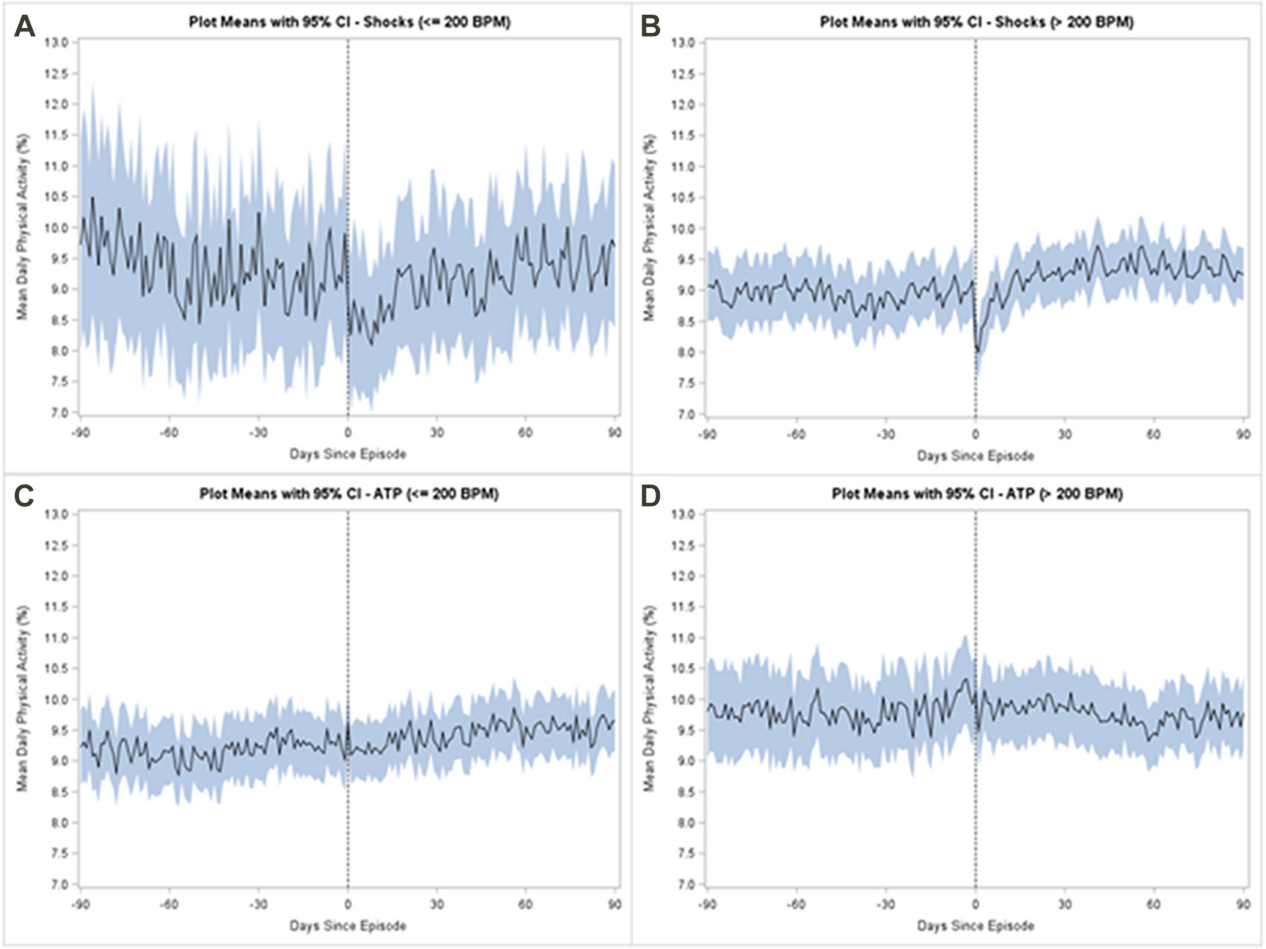
Changes in mean daily physical activity pre- and post-therapy for (A) events ≤200 beats/min with shocks, (B) events >200 beats/min with shocks, (C) events ≤200 beats/min treated with antitachycardia pacing (ATP) only, and (D) events >200 beats/min treated with ATP only. BPM = beats/min; CI = confidence interval.

When comparing the daily CIED-derived activity levels at baseline with the activity level immediately prior to the VA events, we did not find a significant difference in activity for patients with VA events ≤200 beats/min or >200 beats/min with shock or ATP alone (Figures 1 and 2, Table 2).

**Table 2:** Change in daily activity pre- and post-therapy vs baseline by treatment zones and ATP vs shock

Time	Total population	Difference (%)	*P* value (Wilcoxon signed rank test)
≤200 beats/min with 1 or more shocks			
Pre to post	114	–5.8 (–29.5 to 12.3)	.0025∗
Pre to baseline	102	–0.4 (–15.0 to 20.9)	.1655
Baseline to post	100	–6.8 (–25.4 to 8.5)	.0198∗
≤200 beats/min with ATP alone			
Pre to post	615	–1.1 (–12.5 to 13.2)	.7885
Pre to baseline	521	–0.7 (–15.2 to 11.8)	.2633
Baseline to post	546	–3.3 (–17.3 to 13.5)	.0288∗
>200 beats/min with 1 or more shocks			
Pre to post	593	–8.7 (–24.6 to 7.3)	<.0001∗
Pre to baseline	552	0.0 (–16.0 to 14.3)	.6903
Baseline to post	553	–8.8 (–28.8 to 7.1)	<.0001∗
>200 beats/min with ATP alone			
Pre to post	519	–1.8 (–14.3 to 10.7)	.0684
Pre to baseline	447	0.0 (–12.8 to 15.9)	.1098
Baseline to post	472	–2.7 (–16.0 to 16.7)	.8533

Values are n or median (interquartile range).

ATP = antitachycardia pacing.

∗ Significant *P*-value <.05.

#### Changes in activity after VAs

**Figure 3:**
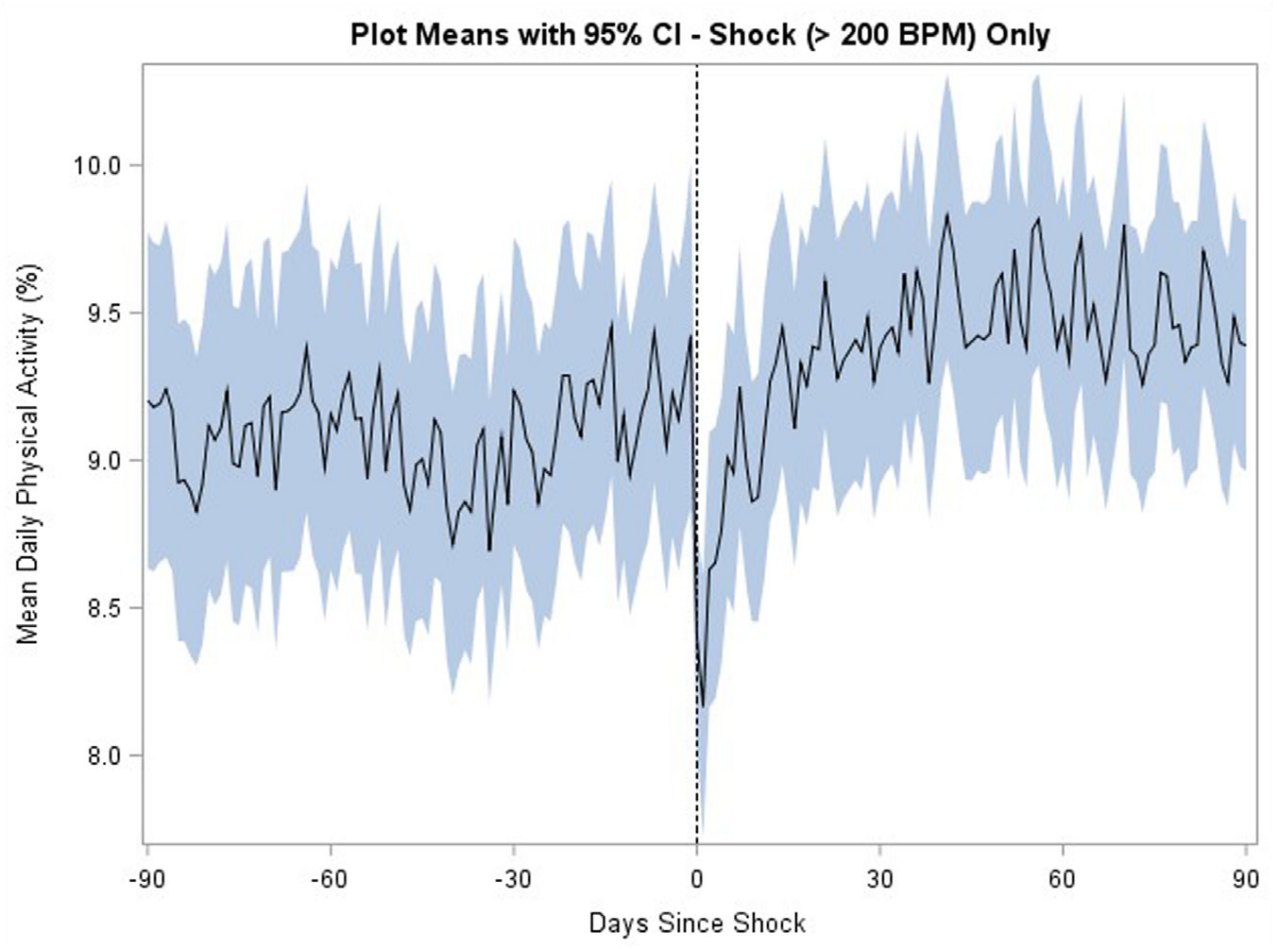
Changes in mean daily physical activity during 90-day pre- and 90-day post-therapy period for events >200 beats/min treated with shock . Activity is reported as percent of day patient is physically active. BPM = beats/min; CI = confidence interval.

On the other hand, patients with VA events >200 beats/min treated with shock (Figure 3) had a significant reduction in activity immediately following a VA event with a median 8.7% reduction (IQR –24.6% to 7.3%, *P <* .001) (Table 2). Similarly, VAs ≤200 beats/min treated with shock were also associated with a reduction in activity following the VA event (–5.8%, IQR –29.5% to 12.3%, *P =* .003).

However, this association was not present for VA events >200 beats/min and VA events <200 beats/min treated with ATP alone (Figure 3, Table 2). A significant change in activity post–VA event compared with both baseline and pre-event activity was seen for all therapy and event types except those ≤200 beats/min with ATP alone, those ≤200 beats/min with no therapy, those ≤200 beats/min overall, and those >200 beats/min with ATP alone (Table 2).

### Case-crossover, sensitivity analysis

Alternate analysis with a case-crossover model was performed in 2503 patients who were not missing a Home Monitoring transmission during one of the analyzed periods (Figure 1).

Patient demographics for this cohort were similar to the overall cohort with similar distribution of ICD and CRT-D devices (Table 3). Importantly, the case-crossover analysis showed a similar change postactivity in an adjusted model as in the original analysis (*P =* .0579) (Table 4).

**Table 3:** Patient demographics for the case-crossover analyses

Characteristic	All patients (n = 2503)	1 or more shocks		ATP alone	
		≤200 beats/min (n = 69)	>200 beats/min (n = 398)	≤200 beats/min (n = 391)	>200 beats/min (n = 373)
Age at enrollment, y	67.0 ± 11.4 (14.7–95.8)	68.2 ± 11.0 (32.1–89.4)	68.1 ± 10.1 (30.5–88.4)	71.0 ± 9.1 (45.4–90.9)	67.3 ± 11.3 (14.7–92.8)
Sex					
Female	606 (24.2)	12 (17.4)	73 (18.3)	73 (18.7)	68 (27.4)
Male	1750 (69.9)	54 (78.3)	288 (72.4)	300 (76.7)	172 (69.4)
Unknown	147 (5.9)	3 (4.4)	37 (9.3)	18 (4.6)	8 (3.2)
Implant type					
ICD	1560 (62.3)	43 (62.3)	238 (59.8)	224 (57.3)	221 (59.3)
Single chamber	189 (7.6)	4 (5.8)	34 (8.5)	18 (4.6)	28 (7.5)
Dual chamber	848 (33.9)	25 (36.2)	118 (29.7)	157 (40.2)	111 (29.8)
DX	523 (20.9)	14 (20.3)	86 (21.6)	49 (12.5)	82 (22.0)
CRT-D	943 (37.7)	26 (37.7)	160 (40.2)	167 (42.7)	152 (40.8)
CRT-DX	10 (0.4)	0 ()	5 (1.3)	0 (0)	2 (0.5)

Values are mean ± SD (range) or n (%).

ATP = antitachycardia pacing; CRT-D = cardiac resynchronization therapy defibrillators; CRT-DX = single-lead cardiac resynchronization therapy defibrillator; DX = single-lead implantable cardioverter-defibrillator system; ICD = implantable cardioverter-defibrillator.

**Table 4:** Unadjusted and adjusted case-crossover analyses

	Physical activity							
	Unadjusted model				Adjusted model (stratified—age/sex/implant)			
	Estimate	Odds ratio	95% CI	*P* value	Estimate	Odds ratio	95% CI	*P* value
Overall	–0.00147	0.999	0.997–1.000	.0712	–0.00203	0.998	0.996–1.000	.0153
Group split								
1 or more shocks	–0.00155	0.998	0.994–1.002	.452	–0.00281	0.997	0.993–1.001	.1955
1 or more shocks + >200 beats/min	–0.00346	0.997	0.992–1.001	.1202	–0.00445	0.996	0.991–1.000	.0579
1 or more shocks + ≤200 beats/min	0.01124	1.011	1.000–1.023	.052	0.00811	1.008	0.996–1.020	.1795
ATP	0.00239	1.002	0.999–1.006	.1465	0.00234	1.002	0.999–1.006	.1616
ATP + >200 beats/min	0.00343	1.003	0.999–1.008	.1521	0.0032	1.003	0.998–1.008	.1867
ATP + ≤200 beats/min	0.00145	1.001	0.997–1.006	.5172	0.00156	1.002	0.997–1.006	.498
No therapy	–0.00301	0.997	0.995–0.999	.0042	–0.00362	0.996	0.994–0.998	.0008
No therapy + >200 beats/min	–0.00613	0.994	0.988–0.999	.0278	–0.00697	0.993	0.987–0.999	.0155
No therapy + ≤200 beats/min	–0.00246	0.998	0.995–1.000	.0312	–0.00303	0.997	0.995–0.999	.0098

ATP = antitachycardia pacing; CI = confidence interval.

#### Association between activity and VA events with no therapy

We performed additional analysis assessing patients with VA events without treatment. Clinical characteristics of this cohort are shown in Supplemental Table 1. In patients with VA events >200 beats/min with no therapy, we showed a significant reduction of activity following the event (Supplemental Table 2, Supplemental Figure 1). Additionally, we found a significant decrease activity in VA events <200 beats/min with no therapy pre– vs post–VA event (Supplemental Table 2, Supplemental Figure 1). Case-crossover analysis showed a significant change in activity post-VA in VA events ≥200 beats/min and VA events <200 beats/min with no therapy (Table 4).

## Discussion

In this large registry study, we report important findings regarding the effects of ICD shock on CIED-derived physical activity but no association with ATP therapy. We show several findings: (1) there were no significant changes in activity in the 7 days prior to a VA event, (2) physical activity was significantly reduced in patients following VA events >200 beats/min treated with an ICD shock, (3) patients with VA events < 200 beats/min did not have any changes in activity following the VA event, and (4) we have shown a significant reduction of activity in patients following VA events >200 beats/min with no therapy.

Recent technological advances facilitate the analysis of activity data to better understand individual responses to device-based therapies and potentially guide individualized treatment decisions that could avoid hospitalization and adverse outcomes.^10^^,^^11^ However, there is a paucity of data comparing temporal changes in activity associated with 2 distinct ICD therapeutic modalities: (1) ICD shock and (2) ATP and potential post-therapy adverse effects.

Prior studies established the feasibility and the usefulness of collecting accelerometer-acquired physical activity data in large cohorts with great overall compliance of over 93.3% of the participants providing sufficient data for activity analysis.^5^ In our study, in a large cohort of patients with ICD/CRT-D devices, we have been able to record and analyze activity in all of those who had Home Monitoring data transferred (100%). Another study reported on CRT patients followed on remote monitoring and suggested that changes in activity following CRT are linked to outcomes^3^; however, they did not report on temporal changes related to VAs.

Contrary to other studies, our registry data provide with a unique opportunity of analyzing a very large sample of activity data and relate it to VAs over time. In addition, the Home Monitoring system transmits data on physical activity on a daily basis. As a result of this, in our study, we have a cumulative follow-up of 5.6 million days of transmission from 18,432 patients.

The most interesting finding of our study with potential clinical implications is the significant decline in physical activity in patients with a VA event >200 beats/min treated with an ICD shock but not ATP therapy. ICD shocks have been reported to be associated with an increased mortality. However, the mechanism for this association is not known. A decline in activity following an ICD shock could either be a contributing factor or the mechanism itself.

In addition, ICD shocks have been correlated with psychological trauma and anxiety in prior studies.^12^, ^13^, ^14^ In one of our Multicenter Automatic Defibrillation Implantation Trial Cardiac - Resynchronization Therapy (MADIT-CRT) substudy, we assessed anxiety levels using the Florida Shock Anxiety Scale (1–10 score) in patients with appropriate or inappropriate shocks or ATP compared with those with no ICD therapy.^15^ We found that ≥2 appropriate or inappropriate shocks were associated with more anxiety despite no changes in the assessment of global quality of life by the EQ-5D questionnaire.^15^ The current study expands our knowledge and findings and suggests that not only anxiety, but also activity levels change following an ICD shock.

The reduction in activity seen after an ICD shock could also happen due to a variety of other factors, including patient discomfort from the shock, concomitant deterioration of functional status due to heart failure and/or recurrent sustained arrhythmias, and concurrent acute noncardiac illness. The fact that shock therapy, in contrast to ATP, may be associated with a significant decrease in activity, suggests that the type of therapy itself adversely impacts patients. It is also possible that patients with an ICD shock more frequently have healthcare utilization or be hospitalized, as reported by Turakhia and colleagues,^16^ and they are inactive during such a time period.

We also did not report significant changes in activity immediately prior to a VA event, potentially suggesting that monitoring in activity in these patients will not help us predict a VA event. However, many studies reported a reduction in activity in patients developing heart failure events.^7^^,^^17^^,^^18^ The lack of predictive value of activity for VA events preclude this variable to be used in clinical models and decision making.

A decrease in postevent activity occurring without device-based therapy provides important information to suggest that ICD therapy itself is unlikely to be the sole cause of the activity reduction. If so, then there may be other underlying contributing factors not yet identified. Our results raise interesting questions regarding the effect of ICD therapy vs no therapy for CIED-detected VA events on subsequent physical activity and the contributions of arrhythmia itself to the activity reduction. The utilization of CIED-derived activity as a digital biomarker may improve our understanding of the potential effects of device-based therapies.

The observed effects of ICD therapy and biphasic energy shock vs ATP on subsequent physical activity have important clinical implications and suggest that monitoring CIED-derived daily activity is helpful to provide additional insights into the physiologic deleterious of ICD shocks. In addition, CIED-derived daily activity could be a useful marker for identifying a high-risk cohort with negative effects of ICD shocks and prompt more aggressive treatment. However, such monitoring of daily activity and the potential effects of intensified therapy for this cohort will need to be prospectively evaluated in future studies.

### Strengths and limitations

The main strength of this analysis is the daily recording of activity levels in a large number of patients and assessing changes immediately prior to and after arrhythmia events. Such analyses could identify smaller and temporal changes in activity not normally detected in clinical studies with longer follow-up periods. Our dataset contains 5.6 million days with transmission. We also performed case-crossover analyses to validate the findings using another method. Another strength is the multitherapy monitoring used with ATP and shock therapy, especially as it relates to temporal changes with prebaseline, baseline, and postbaseline changes in physical activity. However, we disclose some limitations, including the use of 1-axis accelerometer available in Biotronik devices, a sizable number of patients excluded from analyses due to no data available, and the arbitrary definition of baseline, pre-VA, and post-VA periods. In addition, we did not have the possibility to perform electronic health record integration to define patient comorbidities, utilization, or outcomes, and we did not have claims data available. Therefore, we did not have data on hospitalizations following ICD treatments. Although we suggest a strong association between activity and ICD shock, the data sources limit us from ascertaining the mechanism or cause of decreased activity post–VA event shock.

### Conclusion

In this CERTITUDE registry study, we found that CIED-derived daily activity was significantly reduced in patients post–VA events>200 beats/min treated with an ICD shock. However, there were no significant changes in activity immediately prior to a VA event or post–VA events <200 beats/min. CIED-derived daily activity could be a useful marker for assessing the detrimental effects of ICD shocks in CIED patients and could identify those with need for more aggressive treatment.

## Disclosures

Valentina Kutyifa has received grants from Boston Scientific, ZOLL Inc., Biotronik, and the National Institutes of Health; and speaker and consulting fees from Medtronic, Biotronik, ZOLL Inc., and Abbott Medical. Steven Mullen and Camden Harrel are employees of Biotronik. The other authors have no conflicts to disclose.
