# Supplementary material for: Temporal changes in device-derived daily activity related to ventricular arrhythmias from the CERTITUDE registry
Source: Heart Rhythm O2. 2024 Aug 20;5(11):805–12. doi: 10.1016/j.hroo.2024.07.020 (PMC11624418; doi:10.1016/j.hroo.2024.07.020)
Supplement: Supplementary Materials [file mmc1.docx]

**Supplementary Materials**

Supplementary Figure 1. Changes in mean daily physical activity pre- and post-therapy for events with no therapy

**Supplementary Figure 1. Changes in mean daily physical activity pre- and post-therapy for events with no therapy**


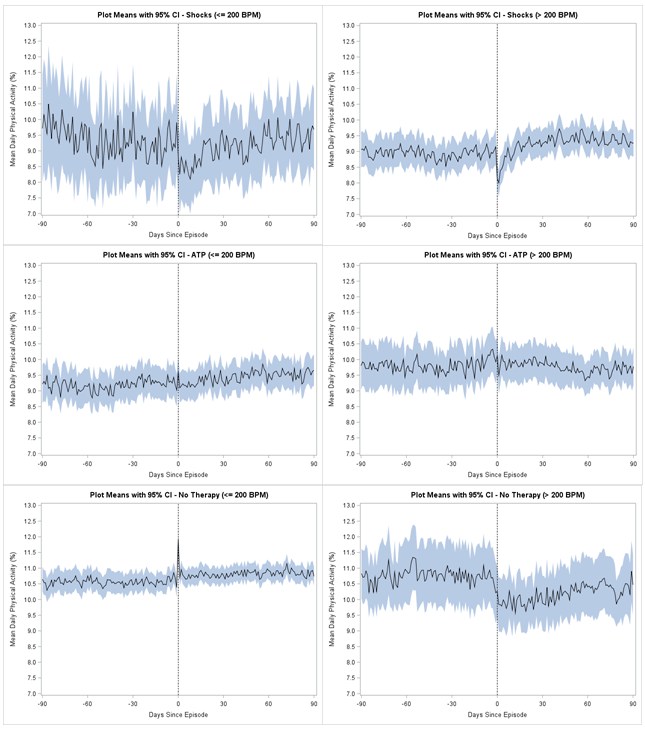


**Supplementary Table 1.** **Baseline demographics for the overall cohort of patients with data on daily physical activity for the no therapy and combined groups**

| **Characteristics** | **No Therapy** | |
| --- | --- | --- |
|  | **≤200 BPM** | **>200 BPM** |
|  | **N = 2568** | **N = 427** |
| **Age at enrollment, years** |  |  |
| Mean, ± SD | 62.1 ± 12.8 | 67.1 ± 11.7 |
| Range | 13.7 to 95.8 | 14.7 to 92.8 |
| **Gender, n (%)** |  |  |
| Male | 1673 (65.1%) | 281 (65.8%) |
| Female | 690 (26.9%) | 117 (27.4%) |
| Unknown | 205 (8.0%) | 29 (6.8%) |
| **Implant Type, n (%)** |  |  |
| ICD | 1825 (71.1%) | 248 (58.1%) |
| Single chamber | 258 (10.1%) | 42 (9.8%) |
| Dual chamber | 894 (34.8%) | 136 (31.9%) |
| DX | 673 (26.2%) | 70 (16.4%) |
| CRT-D | 743 (28.9%) | 179 (41.9%) |
| CRT-DX | 10 (0.4%) | 1 (0.2%) |

**Supplementary Table 2. Changes in mean daily physical activity pre- and post-therapy for events with no therapy and combined groups**

| **Time** | **Total Population** | **Median % Difference (IQR)** | **p-value (Wilcoxon signed rank test)** |
| --- | --- | --- | --- |
| **>200 BPM with No Therapy** | | | |
| **Pre to Post** | 297 | -3.0 (-17.2 %;11.6 %) | 0.0502 |
| **Pre to Baseline** | 262 | -1.0 (-11.9 %;16.3 %) | 0.5234 |
| **Baseline to Post** | 286 | -4.7 (-21.2 %;14.3 %) | **0.0167** |
| **<=200 BPM with No Therapy** | | | |
| **Pre to Post** | 2077 | 0.0 (-10.9 %;14.5 %) | 0.0006 |
| **Pre to Baseline** | 1520 | 0.0 (-13.4 %;14.3 %) | 0.5374 |
| **Baseline to Post** | 1584 | 0.0 (-14.3 %;16.2 %) | 0.1341 |
